# Pairs of Mutually Compensatory Frameshifting Mutations Contribute to Protein Evolution

**DOI:** 10.1093/molbev/msac031

**Published:** 2022-02-08

**Authors:** Dmitry Biba, Galya Klink, Georgii A Bazykin

**Affiliations:** 1 Center of Life Sciences, Skolkovo Institute of Science and Technology, Moscow, Russia; 2 Institute for Information Transmission Problems of the Russian Academy of Sciences (Kharkevitch Institute), Moscow, Russia

**Keywords:** frameshifting indels, evolution of novelty, compensatory evolution

## Abstract

Insertions and deletions of lengths not divisible by 3 in protein-coding sequences cause frameshifts that usually induce premature stop codons and may carry a high fitness cost. However, this cost can be partially offset by a second compensatory indel restoring the reading frame. The role of such pairs of compensatory frameshifting mutations (pCFMs) in evolution has not been studied systematically. Here, we use whole-genome alignments of protein-coding genes of 100 vertebrate species, and of 122 insect species, studying the prevalence of pCFMs in their divergence. We detect a total of 624 candidate pCFM genes; six of them pass stringent quality filtering, including three human genes: *RAB36, ARHGAP6*, and *NCR3LG1.* In some instances, amino acid substitutions closely predating or following pCFMs restored the biochemical similarity of the frameshifted segment to the ancestral amino acid sequence, possibly reducing or negating the fitness cost of the pCFM. Typically, however, the biochemical similarity of the frameshifted sequence to the ancestral one was not higher than the similarity of a random sequence of a protein-coding gene to its frameshifted version, indicating that pCFMs can uncover radically novel regions of protein space. In total, pCFMs represent an appreciable and previously overlooked source of novel variation in amino acid sequences.

## Introduction

The origin of radically novel amino acid sequences remains somewhat of an enigma. Numerically, by far the most common event in evolution of protein-coding genomic segments is a single-nucleotide substitution (Murphy et al. 2004). Although gradual accumulation of such substitutions can modify the characteristics, structure and, in some instances, function of the encoded protein, they only allow reaching a limited neighborhood of the existing sequence in the sequence space (Povolotskaya and Kondrashov 2010). This leaves the question of the possibility of origin of new segments of protein-coding sequences from scratch. Several mechanisms for such events are known. Some involve acquisition of new DNA segments within protein-coding regions, by repeat expansion (Hancock and Simon 2005) or nonrepeat-associated insertions with lengths divisible by 3. Others involve redefinition of an existing DNA segment as protein-coding; these include emergence of exonic segments from intronic ones (Artamonova and Gelfand 2007), sequences encoding N- or C-termini of proteins from 5′- or 3′-UTRs (Vakhrusheva et al. 2011), or even entire genes from noncoding DNA (Neme and Tautz 2013; Bornberg-Bauer et al. 2021); or acquisition of an alternative reading frame for an existing protein-coding sequence (Keese and Gibbs 1992).

Because a previously noncoding sequence is expected to carry an in-frame stop codon roughly every 20 codons, radically new protein-coding sequences are seldom long. Nevertheless, the complete novelty of the encoded sequence may result in reaching remote domains of the sequence space, which may be then fine-tuned in the course of subsequent evolution (Carvunis et al. 2012).

Another possible mechanism of origin for a new amino acid-coding sequence is frameshifting insertions and deletions (hereafter, indels). Generally, they are considered to be an unlikely source of novelty (Ohno 1970) because they usually cause major disruptions in the amino acid sequence and its truncation through gain of premature stop-codons, and therefore should be extremely rare to fix due to fitness cost associated with them. Nevertheless, there are some examples of fixation of frameshifting indels and, though the protein function is often lost in these cases, sometimes it is retained (Hahn and Lee 2005). Moreover, in some cases such indels give rise to a totally new protein function (Ohno 1984; Vandenbussche et al. 2003). Still, these mutations tend to fix only if they happen near the 5′- or 3′-end of a gene, which likely lowers their impact on protein structure (Hu and Ng 2012; MacArthur et al. 2012).

Another factor which may prevent a frameshifting indel from disrupting overly long segments of protein-coding sequence is a gain of a second compensatory indel in close proximity to the first one. If the total length of the two indels is divisible by 3, the reading frame will be restored and the only amino acids changed would be those between these indels. In experimental evolution of phages, indels are frequently compensated by frame-restoring indels—a finding which has been instrumental in the discovery of the structure of genetic code (Crick et al. 1961). However, the role of compensatory pairs of indels in natural evolution has not been studied systematically. Meanwhile, there are some reasons to believe that the changes to protein structure during frameshifts are not as substantial as replacement with a totally random sequence: some key physicochemical properties of the encoded amino acid sequence tend to be preserved by frameshifts (Wang et al. 2016; Bartonek et al. 2020). Finally, the intermediate, yet-to-be-compensated state of a protein may not be as deleterious as one could have expected, since ribosomes often bypass premature stop-codons, which are the most detrimental feature of such state (Rockah-Shmuel et al. 2013). With all these considerations taken into account, we suggest that pairs of mutually compensatory frameshifting mutations (hereafter, pCFMs, pCFM for a single such pair) may be of importance in the evolution of novel amino acid sequences.

In this paper, we systematically study pCFMs that comprised indels that have occurred rapidly one after the other in the evolutionary history of vertebrate and insect genes. Using a phylogenetic reconstruction of ancestral states, we describe multiple instances of such events, study the characteristics of the proteins and protein segments in which they occur, and predict their impact on the properties of the encoded protein sequence.

## Results

### Detected Pairs of Compensatory Frameshifting Mutations

We designed a parsimony-based algorithm that infers pCFMs from a multiple sequence alignment of a gene and the corresponding phylogenetic tree (see “Inference of pCFMs” section in Materials and Methods). Inference of frame-disrupting indels from interspecies comparisons of genomic sequences is complicated by a low signal-to-noise ratio due to sequencing, assembly, or alignment errors. We focused on those pCFMs that are most likely to be reliable as follows. Firstly, we reasoned that selection against a single bona fide frame-disrupting indel is generally expected to be strong, and therefore those indels that have apparently survived for long periods of evolutionary time are more likely to be artifactual. Therefore, we considered only those pairs of indels that both occurred on the same segment of the phylogenetic tree, which means that the possible intermediate state (i.e., that carrying just one of the indels but not the other) was not observed in any extant species or internal phylogenetic nodes. Secondly, we reasoned that shorter indels are less likely to be artifactual; therefore, we only considered indels of one or two nucleotides in length. Although some artifactual indels can still slip through these filters, we expect this set to be strongly enriched in true pCFMs.

**Fig. 1.**
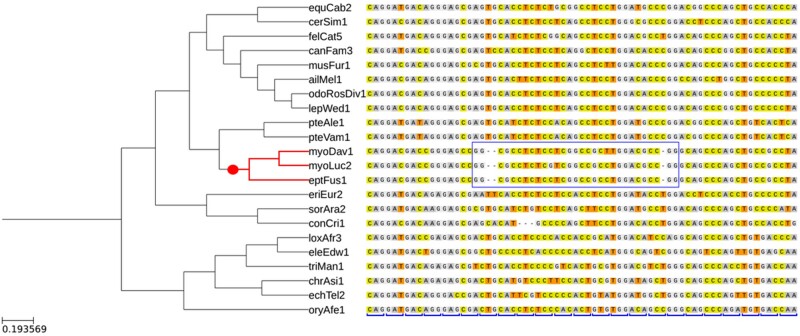
An example of a pCFM: a fragment of alignment of the *RASSF4* gene, one of the six identified genes carrying high-confidence pCFM. At the left, a cladogram of the corresponding species is shown, with the pCFM-carrying clade marked in red, and a subset of the species devoid of the pCFM, in black (some of the non-pCFM-carrying species are not shown). The reconstructed phylogenetic position of the pCFM is marked with a red dot. The region with the shifted reading frame is marked by a blue rectangle. The original reading frame is indicated at the bottom of the alignment by blue square brackets.

Under these restrictions, we found a total of 624 genes (468 in vertebrates and 156 in insects) that carried pCFMs. We refer to this set of genes as the “low-confidence data set.” Both vertebrate and insect data sets show a deletion bias: the percentage of deletions among pCFM-forming indels was 0.58 and 0.66, respectively. The vast majority of pCFMs were only observed in a single species each; multiple species inheriting the same pCFM were observed only for 15 genes in vertebrates and for 12 genes in insects (table 1). Among them, six vertebrate genes and five insect genes were described in multiple databases and/or were supported by mRNA or protein sequence data (supplementary table 2, Supplementary Material online; see the “Postfiltering of pCFMs” section in Materials and Methods). These genes were then realigned with four widely used aligners—muscle (Edgar 2004), clustalw (Higgins and Sharp 1988), prank (Löytynoja 2014), and t-cofee (Notredame et al. 2000)—on top of the initial mafft alignment. We discarded those genes that did not contain compensatory indels in at least one of these realignments, leaving us with five genes in vertebrates and one gene in insects (fig. 1 and table 2). We refer to these six genes as the “high-confidence data set.”

**Table 1.** Number of Genes Surviving Each of the Filtering Stages.

	Vertebrates	Insects
Number of analyzed protein-coding genes	21,208	15,283
Number of genes with pCFM comprised indels of length ≤2 (low-confidence data set)	468	156
Number of genes with multiple species carrying pCFM	15	12
Number of genes with multiple species carrying pCFM that passed all the quality filters (high-confidence data set)	5	1

**Table 2.** The Six pCFM-Carrying Genes in the High-Confidence Data Set.

	Gene Name	Number of Species with pCFM^a^	Number of Nucleotides between Indels in pCFM	Indel Types (5′, then 3′)
Vertebrates	*RAB36* ^b^	11	99	2-bp deletion, 1-bp deletion
	*ARHGAP6* ^b^ ^,^ ^c^	9	7	1-bp deletion, 2-bp deletion
	*NCR3LG1* ^b^ ^,^ ^c^	4	32	1-bp deletion, 1-bp insertion
	*RASSF4*	3	26	2-bp deletion, 1-bp deletion
	*SPATA24* ^d^	1	14	1-bp deletion, 1-bp insertion
Insects	*Wds*	2	10	2-bp deletion, 1-bp deletion

Note.—The number of species carrying the pCFM (^a^) includes the species that were parts of the postframeshift clades, but that were excluded at the filtering step (see the “Inference of pCFMs” section in Materials and Methods). For *SPATA24*, a closely related pCFM-carrying species (*Cavia aperea*) not present in the original alignment was found in ENSEMBL; therefore, this gene was included in the final list. See supplementary table 1, Supplementary Material online, for lists of pCFM-carrying species for each pCFM.

b Both ancestral and derived state confirmed by mRNA transcripts.

c Derived state confirmed by protein sequence.

d Ancestral state confirmed by protein sequence.

A previous work (Hu and Ng 2012) has identified a pCFM between human and dog in the FLJ43860 gene. However, we were unable to confirm this finding, as the corresponding amino acid-coding sequence is not detected by TBlastN in the reference human genome, and this gene is currently annotated as a pseudogene in HGNC gene symbol report (Povey et al. 2001).

### pCFMs Are Overrepresented Near Gene Ends

Uncompensated indels with lengths not in multiple of 3 occur more frequently near ends of coding sequence, probably because the deleterious effect of a frameshift is weaker at these positions (Hu and Ng 2012; MacArthur et al. 2012). The pCFMs could follow the same pattern; alternatively, they could be distributed along the sequence uniformly as they do not cause any disruption of the protein-coding potential of the sequence downstream of them.

**Fig. 2.**
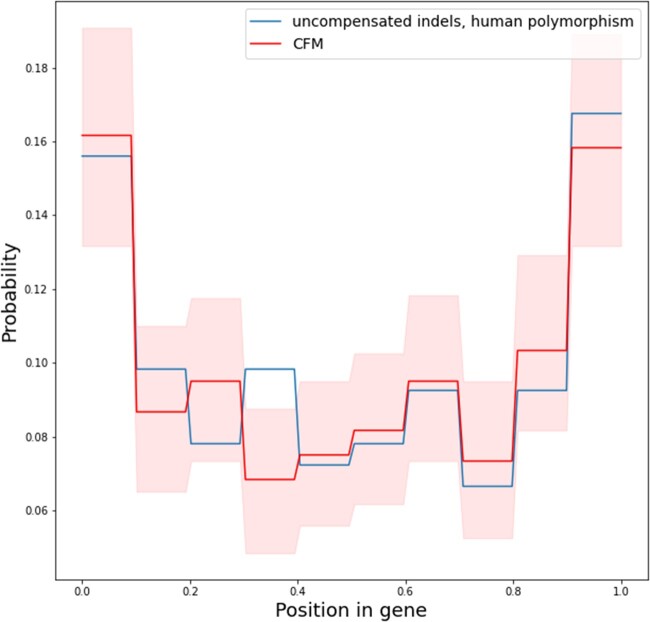
The distribution of uncompensated indels in human polymorphism data (blue line) and pCFMs (red line) along the gene coordinates. Pale red areas correspond to the 95% bootstrap confidence intervals. The horizontal axis indicates the position along the length of the coding sequence, with 0.0 corresponding to its start, and 1.0, to its end.

We find that the pCFMs of the low-confidence data set are biased toward gene ends (fig. 2). To quantify this, we compared the distribution of pCFM positions along the CDS with two other distributions: a uniform distribution, and that of uncompensated indels in human polymorphism data obtained from Ng et al. (2008) (fig. 2). Specifically, we obtained the relative position of the pCFM along the CDS sequence as $P=\frac{p_{1}}{L-\left(p_{2}-p_{1}\right)}$, where $p_{1}{,p_{2}}^{}$ are the positions of the 5′ and the 3′ indels and L is the length of the gene; P=0 when the 5′ indel is positioned at the 5′-most end of the CDS, and P=1 if the 3′ indel is positioned at the 3′-most end of the CDS. The distribution of P within a coding sequence significantly deviates from the uniform distribution (Kolmogorov–Smirnov test, *P* = 0.001), but does not differ from the distribution of indels in human polymorphism (Kolmogorov–Smirnov test, *P* = 0.12).

### Genes with Compensatory Frameshifts Are Less Conserved

As frameshifts disrupt the protein structure, we hypothesized that they occur more frequently in those genes that are young, dispensable, and/or less conserved in their sequence.

To test this hypothesis, first, we estimated the negative selection that had acted on the sequence of pCFM-carrying genes in the course of their evolution between species. For this, we measured the ratio of nonsynonymous to synonymous substitution rates *ω* for the pCFM-carrying genes, and compared it with the same value calculated for a set of genes without a pCFM. This control set was compiled in such a way as to ensure that a pCFM could be found in its genes if it were present. Negative selection is relaxed in pCFM-carrying genes for the low-confidence data set (Wilcoxon signed rank test, WSRT *P* = 0.025), although this result was not robust to the list of species compared with calculate *ω* (supplementary table 4, Supplementary Material online). No such difference was observed in the high-confidence data set (Kolmogorov–Smirnov test for uniform distribution of individual *P* values, KST *P* = 0.303, supplementary table 4, Supplementary Material online).

Second, we asked whether the pCFM-carrying genes were more dispensable in interspecies evolution, that is, if they were more likely to be lost in a fraction of species. For this, we counted the fraction of species in our alignment for which the copy of this gene was likely functional, that is, had both a start and a stop codon, the number of nucleotides divisible by 3, and no inframe stop codons. We found that the pCFM-carrying genes of the low-confidence data set were, on an average, lost in a higher fraction of species, compared with their counterparts without pCFMs (WSRT *P* = 5.4e-9). Again, no such differences were observed for the high-confidence data set, likely due to the small sample size (KST *P* = 0.277).

Third, we hypothesized that the pCFM-carrying genes are younger than non-pCFM-carrying ones, as the age of the gene also correlates with its conservation (Albà and Castresana 2005). To estimate gene age, we used the distance from the MRCA of species with a working copy of the gene to the phylogenetic tree root. Indeed, pCFM-carrying genes turned out to be unexpectedly young both in the low- and high-confidence data sets (WSRT and KST *P* values equal to 0.004 and 0.028, respectively). Calculating distance to root as number of branches rather than sum of branches length yields the same results.

**Fig. 3.**
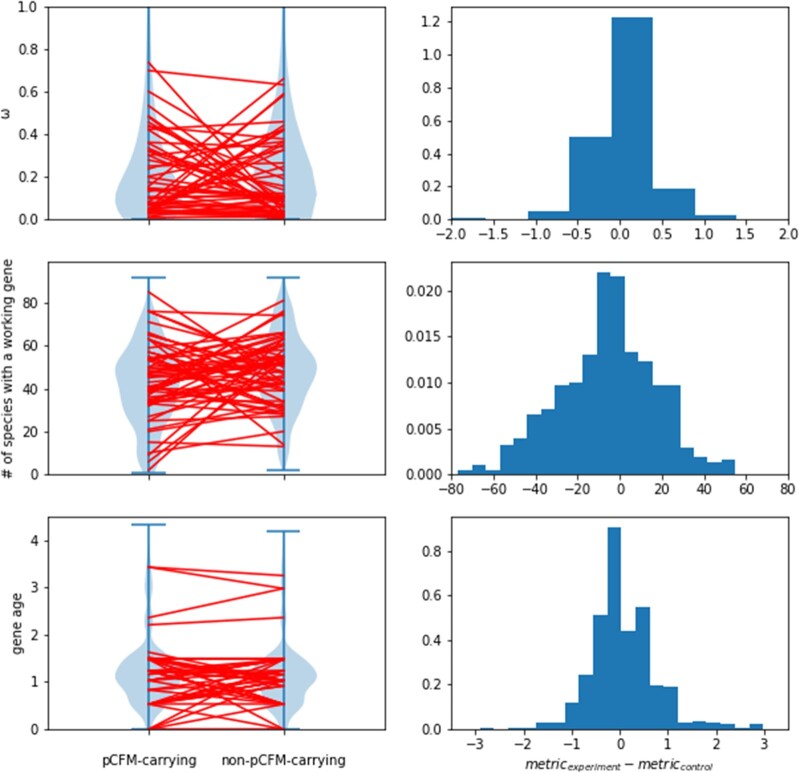
Reduced conservation of pCFM-carrying genes. Each row corresponds to a metric of conservation, from top to bottom: the ratio of nonsynonymous to synonymous substitution rates *ω*, the number of species with a functional copy of a gene, and gene age. On the left, the distributions of these metrics in the low-confidence data set are presented in pCFM-carrying and non-pCFM-carrying genes. These genes are paired, and the red lines represent a random 10% of these pairs. On the right, the distributions of (experiment-control) differences are presented for the same metrics. For *ω* and gene age, these distributions are biased in the positive direction, as higher *ω* and gene age (measured as distance from root to MRCA of species with a functional gene) correspond to lower conservation. For the number of species with a functional gene, the distribution is biased in the negative direction, since lower number of species with a functional gene corresponds to lower conservation.

Overall, pCFM-carrying genes tend to be less conserved, although this effect is not particularly strong (fig. 3).

Next, we hypothesized that the selection on the protein sequence has changed at the time of the pCFM fixation. For example, a disruption of protein structure due to a pCFM could have made subsequent substitutions less deleterious or even beneficial due to epistatic interactions between protein segments.

To test this, for each gene, we fit two *ω* values: one for the clade of the phylogenetic tree descendant to the pCFM-carrying branch, and another for the remaining branches. To test whether this model describes our data better than the model with a single *ω*, we used the likelihood-ratio test. No significant difference was found for any of the genes (table 3); that is, no statistically significant change in the rate of evolution following the pCFM has been observed.

**Table 3.** Selection in pCFM-Carrying Genes under Different Evolutionary Models.

Gene	One-Parameter Model	Two-Parameter Model		Comparison of the Model Fits	
	*ω* for the One-Parameter Model	*ω* for the Non-pCFM-Carrying Branches	*ω* for the pCFM-Carrying Clade	LRT Statistics Value	LRT *P* Value
*RASSF4*	0.166	0.163	0.254	3.78	0.05
*NCR3LG1*	1.23	1.71	0.867	1.76	0.183
*RAB36*	0.229	0.24	0.211	0.22	0.636
*SPATA24*	0.183	0.181	0.284	0.734	0.391
*ARHGAP6*	0.184	0.184	0.187	0.01	0.912
*Wds*	0.013	0.013	0.015	0.128	0.721

Note.—*ω*, the ratio of nonsynonymous to synonymous substitution rates. See text for model details.

### Protein Regions Spanned by pCFMs Are Less Conserved

As shown in the previous section, pCFMs preferentially shift in genes that encode weakly conserved or evolutionarily disposable proteins. Independently, we hypothesized that the pCFMs tend to occur at those regions of the proteins that are weakly conserved, so that the disruption of their structure does not affect protein function.

**Fig. 4.**
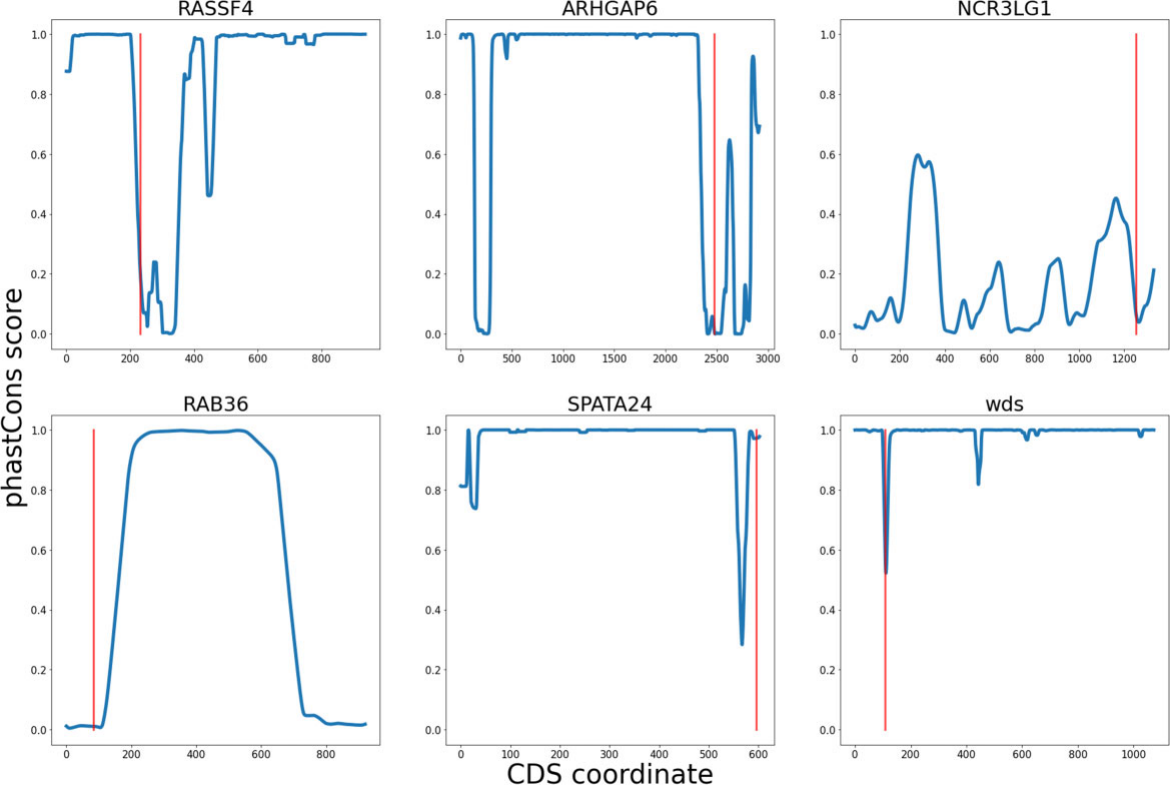
The sequence spanned by a pCFM tends to be located at poorly conserved gene regions. Each panel corresponds to a gene with a pair of compensatory frameshifts. The red line indicated the position of the region spanned by a pCFM. The blue lines show the conservation of the corresponding gene region calculated as the mean phastCons score in a sliding window of length equal to the length of the region spanned by a pCFM.

To test this hypothesis, we compared the amino acid-level conservation of the region spanned by a pCFM with that of other CDS segments of the same gene (fig. 4). In both low- and high-confidence data sets, pCFMs test to reside within less conserved regions as measured by phastCons score (Felsenstein and Churchill 1996) (KST *P* values are 0.037 and 0.001, respectively). This result also holds if the conservation metric used is the Shannon’s entropy for the amino acid site, and is robust to the choice of species for which these metrics are calculated (see supplementary table 4, Supplementary Material online). These results indicate that pCFMs tend to fix in poorly conserved regions of proteins.

### The Effect of pCFMs on the Encoded Amino Acid Sequence

Due to the structure of the genetic code and the characteristics of the structured amino acid sequences, frameshifting mutations generally preserve the physicochemical properties of the encoded protein (Wang et al. 2016; Bartonek et al. 2020). We asked if the pCFMs additionally tend to occur at those positions where this preservation is more precise.

Specifically, we hypothesized that pCFMs tend to fix at positions where they do not substantially affect the structure of the encoded protein according to two similarity metrics. To test this hypothesis, for each of the six pCFM-carrying genes from the high-confidence data set, we calculated the similarity between their amino acid sequences before and after the pCFM (see “Similarity Metrics” subsection in Materials and Methods). We used ancestral state reconstructions (see “Ancestral States Reconstruction” subsection in Materials and Methods) to infer the sequence in which the pCFM has fixed. We asked if the sequence resulting from a pCFM was unexpectedly similar to its ancestral version prior to the pCFM. To calculate the expected distribution of the similarity values, we introduced in silico pCFMs in different genes sampled randomly from the genome, preserving the indel types (insertion/deletion), lengths (1 or 2), and the number of nucleotides between them.

**Fig. 5.**
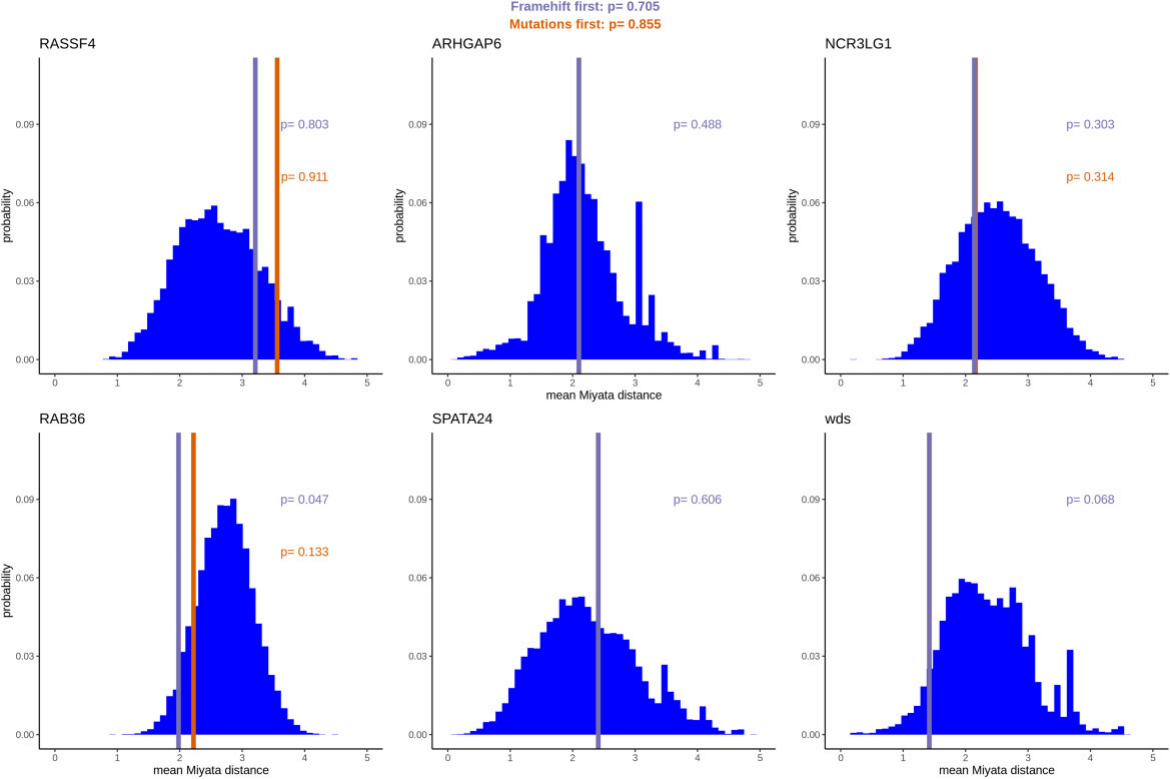
The effect of pCFMs on the physicochemical properties of the encoded amino acid sequence as measured by the mean Miyata distance. The vertical lines represent the Miyata distance between the reconstructed ancestral sequence of the gene immediately prior to the pCFM, and the reconstructed (or observed) derived state of this sequence immediately after the pCFM. The purple and orange lines differ in treatment of single-nucleotide substitutions that have happened on the same phylogenetic branch as the pCFM: such substitutions are assumed to have occurred either after (purple line) or before (orange line) the pCFM; if no such substitutions were present, only the purple line is shown. The blue bars represent the distribution of Miyata distances between each of the 10,000 protein-coding sequences randomly drawn from the genome, and a version of this sequence with a pCFM spaced identically to that in the considered gene. The numbers in each panel correspond to the *P* values obtained as the percentile of the distribution, with low *P* values corresponding to lower-than-expected Miyata distances. The *P* values of Kolmogorov–Smirnov test for uniform distribution of *P* values are shown at the top.

We used two measures of sequence similarity between amino acid sequences: the Miyata distance and differences in hydropathy index. However, we present here only the result for Miyata distance for brevity (fig. 5); all the results on hydropathy index difference can be found in the Supplementary Material online (supplementary figs. 3–5, Supplementary Material online). For individual genes, the observed effect of pCFM on the amino acid sequence matched the expectations, with none of the genes having significantly lower-than-expected differences between the ancestral and derived sequences after the Bonferroni correction. Overall the effect of pCFMs across all genes is also absent (KST, *P* value = 0.093 and 0.164).

Thus, there was no general tendency for pCFMs to preserve the biochemical properties of the amino acid sequences spanned by them to a higher degree than would be expected at a random position of a random protein-coding gene.

### Interaction between pCFMs and Single-Nucleotide Substitutions

Having established the basic properties of the pCFMs, we then asked if they had affected the accumulation of single-nucleotide substitutions. We only perform this analysis for the more reliable high-confidence data set. Admittedly, this analysis has low power due to the scarcity of detected pCFMs, and increasing the sets of analyzed species and/or acquisition of additional support for the currently low-confidence pCFMs could increase its sensitivity.

Conceivably, changes in physicochemical properties of a protein segment caused by a pCFM could be partially compensated by subsequent single-nucleotide substitutions in the region spanned by them, if such substitutions make the protein more similar to the pre-pCFM state. Additionally, a pCFM could preferentially occur in regions where preceding amino acid substitutions made its effect less pronounced. To study this, we analyzed the interactions between pCFMs and substitutions that happened on the same phylogenetic branch in their effect on the characteristics of the encoded amino acid sequence.

**Fig. 6.**
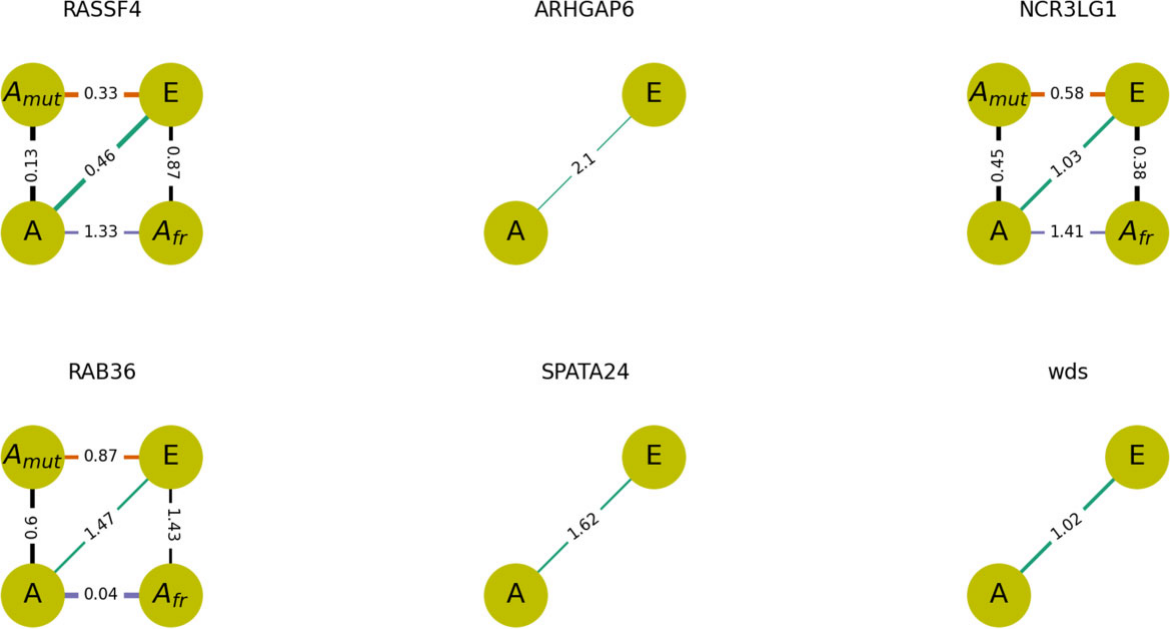
Effects of a pCFM and amino acid substitutions occurring on the same phylogenetic branch as pCFM on the physicochemical properties of the encoded protein, according to the difference in hydropathy of the encoded amino acid sequences. The four circles in each panel represent the four sequences: without pCFM or substitutions (ancestral state, *A*); with substitutions but without pCFM (*A*_mut_); with pCFM but without substitutions (*A*_fr_); with both pCFM and substitutions (derived state, *E*). The numbers on the lines connecting these states represent the distances between corresponding sequences; smaller distances are depicted with bolder lines. Omitted intermediate states (*A*_mut_ or *A*_fr_) correspond to cases when the substitutions are synonymous in the context of pCFM and nonsynonymous in the context of the ancestral state, or vice versa. Both intermediate states are omitted if no amino acid substitutions happened on the considered branch.

In figure 6, we measure the amino acid similarity between the reconstructed ancestral variant of the protein (*A*), the variant carrying pCFM (*A*_fr_), the variant carrying the single-nucleotide substitutions that occurred at the same branch as pCFM (*A*_mut_), and the variant carrying both pCFM and the substitutions (*E*). The observed patterns of pairwise similarity between these variants differed between proteins.

Importantly, we were unable to infer the order in which pCFM and substitutions occurred on the same branch. Therefore, we had to consider two possibilities: that pCFM occurred prior to the substitutions, and that pCFM occurred after the substitutions. We put forth two hypotheses with regard to the similarity patterns: 1) that the substitutions occurred after the pCFM, and compensated for its effect; 2) that the substitutions occurred prior to the pCFM, and made it permissible, so that the effect of the pCFM was weaker on the mutated background.

**Fig. 7.**
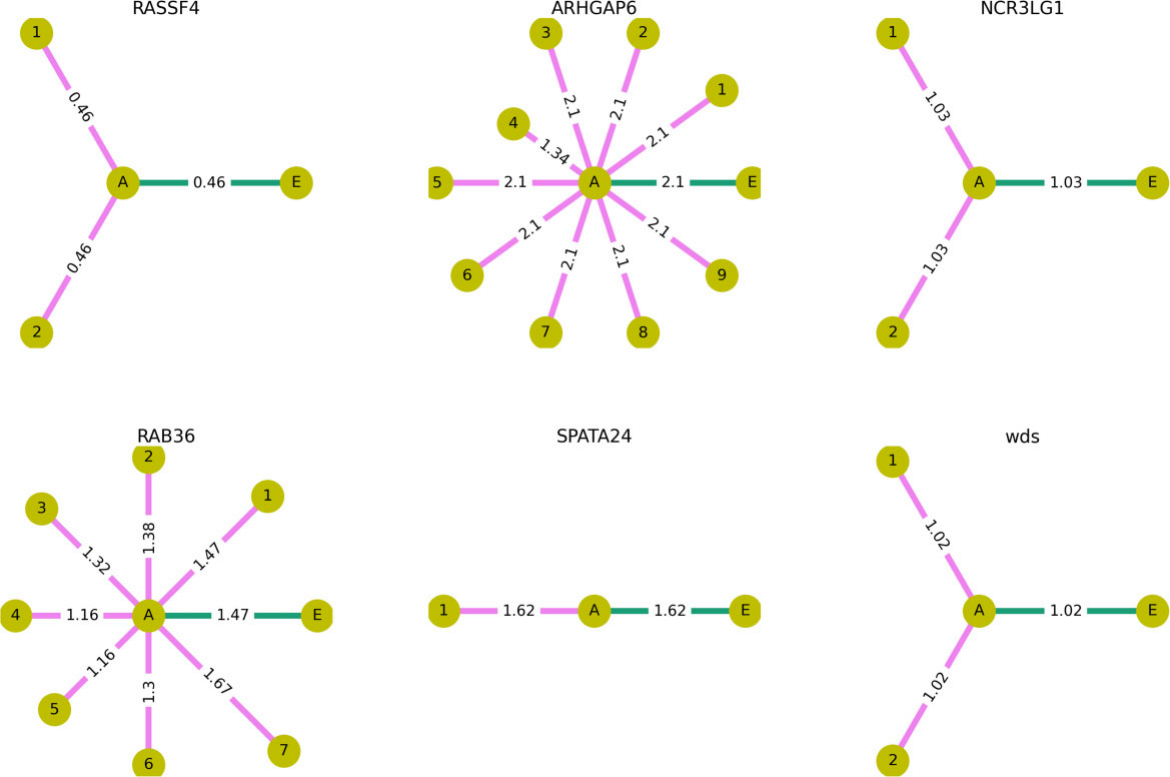
Effects of amino acid substitutions occurring on the phylogenetic branches descendant to pCFM on the physicochemical properties of the encoded protein, according to the Miyata difference between the encoded amino acid sequences. In each panel, the circles correspond to the different states of a sequence: without pCFM (*A*); with pCFM (*E*, always to the right of *A*); and with both pCFM and mutations on the descendant branches (terminal leaves states, denoted by numbers). The lengths of the lines and the numbers on top of them represent distances between the corresponding states; many of the distances are identical, reflective of the substitutions common to the corresponding species.

Under (1), we expect the (*A*, *E*) distance (green in fig. 7) to be lower than the (*A*, *A*_fr_) distance (purple). This was the case for substitutions in *RASSF4* and *NCR3LG1* by hydropathy measurements.

Under (2), we expect the (*A*_mut_, *E*) distance (orange) to be lower than the (*A*, *A*_fr_) distance (purple). This was the case for substitutions in *RASSF4* and *NCR3LG1* by hydropathy measurements.

Nevertheless, we observe no general tendency for the substitutions to be compensatory or permissive. In other words, neither the substitutions that occurred before nor those that occurred after the pCFMs were helpful for the protein to become more like its ancestor.

Next, we hypothesized that the deleterious effect of pCFM can be compensated by substitutions occurring at subsequent branches in the phylogeny (fig. 7). If this hypothesis holds true, we expect the distance between *A* and the terminal descendant branch (magenta in fig. 7) to be smaller than the (*A*, *E*) distance (green). This was the case in *RAB36* by Miyata distance measurements and in *ARHGAP6* and *RAB36* by hydropathy measurements (supplementary fig. 5, Supplementary Material online). Overall, however, we see no tendency for post-pCFM substitutions to be compensatory.

## Discussion

In a recent paper, Bartonek et al. (2020) indicated that frameshifts tend to preserve the physicochemical properties of the encoded amino acid sequence, and asked: “can evidence be found that frameshifting has indeed played a relevant role during evolution of real proteins?” Here, we address this question, and show that this is indeed the case. We describe (table 1) 156 instances in the recent protein evolution of insects, and 468 cases in vertebrates, where a pCFM has fixed in the course of evolution of the lineages that gave rise to extant species. By using stringent filtering criteria, we focus on the one insect and five vertebrate genes for which this evidence is the most robust, and study how they affect the encoded amino acid sequence. Importantly, we focus on pCFMs that happened rapidly one after the other (so that they were observed on the same segment of the phylogenetic tree); thus, we ignore any possible instances of a longer time lag between the two events, including possible cases of resurrection of established pseudogenes (Esfeld et al. 2018).

Among our pCFMs consisting of two same-type events (insertion–insertion or deletion–deletion), the deletion–deletion pairs are more frequent (table 2), and this pattern is also observed in the larger unfiltered data set. This is consistent with the fact that, at least in *Drosophila*, deletion mutations are more frequent than insertion mutations (mutational deletion bias). In functional regions, this bias is compensated by a higher probability of insertions to spread and be fixed by selection (fixational insertion bias) (Leushkin et al. 2013). As a result, among fixed in-frame exonic mutations, insertions and deletions are equally frequent (figure 2 in Leushkin et al. [2013]). The retention of the deletion bias for our fixed pCFMs might suggest that unlike in-frame indels, selection against out-of-frame insertions and deletions is similar, so that the excess of deletions among fixed mutations recapitulates the biases at origin of these mutations.

### pCFMs and Sequence Conservation

How did the two indels causing a pCFM happen in the course of evolution? There are two possibilities: either the two indels happened simultaneously in one generation and fixed in the population, or they happened one after the other. In the latter case, an intermediate uncompensated state should have persisted through generations. What evidence can be used to distinguish between these two scenarios?

The patterns of conservation that we observe are inconclusive to this end. Indeed, we observe that the pCFMs of both the high- and the low-confidence data sets are somewhat biased toward weakly constrained genes, and, within genes, toward weakly constrained segments. Both these patterns can be expected under both above scenarios. Indeed, both the pCFM and the individual frameshifting indels are radical events which are likely to disrupt the structure of the encoded protein, and therefore are expected to be biased toward weakly constrained genes and gene regions.

Perhaps more informative is the distribution of pCFMs along the CDS. We find that, for the larger low-confidence data set, this distribution recapitulates that of uncompensated indels (fig. 2). This suggests that selection has had time to act on the intermediate state (i.e., that defined by a single frameshifting indel). Indeed, as the overall selective constraint is generally rather uniform along the CDS length (Shabalina et al. 2004), the same uniformity is expected for pCFMs if the two indels occur simultaneously. By contrast, if indels occur one after the other, we expect the distribution of pCFMs to be primarily determined by the effects of individual indels rather than their pairs, and therefore to follow the distribution of uncompensated indels, which is the pattern that we observe.

### Expected Frequency of pCFMs

In a functional gene, a single frameshifting mutation may cause a loss in fitness, which can then be compensated by the second frameshifting mutation. There is extensive theoretical literature aiming to estimate the possibility and rates of compensatory evolution under various ranges of population genetics parameters. To our knowledge, the first such model was proposed by Kimura (1985); using diffusion approximation, he showed that the rate of compensatory evolution at tightly linked loci is nonnegligible over a wide range of *N*_e_*s*, where *N*_e_ is the effective population size and *s* is the coefficient of selection disfavoring the intermediate variant. This approach was further developed by Stephan (1996) who focused on RNA evolution and expanded the model assumptions to different selection coefficients against different intermediate mutants.

In subsequent work, two distinct mechanisms for such valley crossing were considered. The first one is sequential fixation of mutations: the first (single or multiple) mutations are deleterious and fixed by drift, and the last mutation is advantageous against their background and is fixed by selection. Under the second mechanism, the intermediate deleterious variant is never fixed; instead, the haplotypes carrying it remain at low frequencies, either due to genetic drift (so-called stochastic tunneling; Iwasa et al. 2004) or deterministically under mutation–selection balance, before they are “rescued” by subsequent compensatory mutations. The range of parameters leading to each regime was explored by Weissman et al. (2009) in asexual populations; for sexual populations, he has shown that low recombination rates may even facilitate valley-crossing (Weissman et al. 2010). One of the results of this study was that, quite intuitively, tunneling is relevant for larger *N*_e_, whereas sequential fixations are relevant for smaller *N*_e_. Carter and Wagner (2002) also obtained similar results, invoking differences in *N*_e_ to explain the differences in compensatory evolution rates of enhancers in vertebrates compared with *Drosophila*.

We adopt a simplistic model to estimate the number of pCFMs we could expect to find in vertebrate and insect genome-scale data sets in the presence or absence of an intermediate uncompensated state. To model the evolution through an intermediate state, we consider two tightly linked positions such that mutations are deleterious in each of them separately but are neutral in combination. The selection coefficient against both single mutants s is assumed to be the same and to equal that against a loss of function in a nonessential gene. This selection is assumed to be strong, such that individual frameshifting mutations are unlikely to fix in a population under realistic *N*_e_. Therefore, we only consider the rate of tunneling, assuming that the first frameshifting mutation is maintained by mutation–selection balance before the second, compensatory mutation occurs. These assumptions are close to those of Kimura’s (1985).

Let L be an average gene length; $p_{1}$, the rate of the first uncompensated mutation per nucleotide per generation; and $p_{2}$, the rate of the second, compensatory mutation per nucleotide per generation. The probability of a frameshifting mutation at some position of this gene per generation *m*_1_ then equals:

$$m_{1}=1-{\left(1-p_{1}\right)}^{L}.$$

Similarly, the probability of a compensatory mutation at some position of this gene per generation *m*_2_ equals:

$$m_{2}=1-{\left(1-p_{2}\right)}^{l},$$

where l is the number of nucleotides surrounding the first mutation where the second mutation would be compensatory.

Under mutation–selection balance (Crow and Kimura 1970), the expected frequency of uncompensated frameshift in a population equals $m_{1}/s$. The compensated genotypes will then originate by mutation of the deleterious alleles at per generation rate of $m_{1}m_{2}/s$.

If compensation is exact, so that the compensated genotype has the same fitness as the ancestral variant, the rate of fixation of such compensatory pairs equals the rate of the mutation giving rise to them: $P_{\mathrm{fix}}=m_{1}m_{2}/s$ (Kimura 1983). Over T generations, a total of $Tm_{1}m_{2}/s$ such pairs are expected to have occurred.

To estimate $p_{1}$ and $p_{2}$, we consider four types of deleterious frameshifting mutations: insertions of lengths 1 or 2 and deletions of lengths 1 or 2. For each of these mutations, we consider the sets of mutations that can compensate for it (e.g., an insertion of length 1 can only be compensated by an insertion of length 2 or a deletion of length 1 but not by a deletion of length 2), and sum over the probabilities of these mutations. The resulting $P_{\mathrm{fix}}$ terms are the sums over these pairs of deleterious and compensatory mutations.

We use the estimate for the de novo mutation rate $\mu =10{}^{-8}$ for vertebrates from Kong et al. (2012) and $\mu =4.9*10^{-9}$ for *Drosophila* from Assaf et al. (2017); the estimates for $p_{1}$ for each type of frameshifting mutation from Leushkin et al. (2013): 0.020μ, 0.010μ, 0.042μ and 0.015μ for insertion of length 1, insertion of length 2, deletion of length 1, and deletion of length 2, respectively; and the estimate for the selection coefficient against a heterozygous loss of function in nonessential genes s=0.0015 in *Drosophila* (Langley et al. 1981) and s=0.005 for vertebrates (estimated for humans in Cassa et al. [2017]). We estimated T and L from our data. For each gene, T is the mean length of a phylogenetic tree including only the species that have passed all filtering steps, without accounting for the terminal branches (since we only consider indels inherited by multiple species) and measured in the genome-wide number of synonymous substitutions. In our data, T=176,536,337 for vertebrates, and T=403,272,889 for insects. L is the mean length of analyzed genes, and equaled L=1,897 for vertebrates and L=1,204 for insects. We take l to equal 100 (the size of all the regions spanned by observed pCFMs is less than that, table 2).

Under these estimates, we obtain ${Tm}_{1}m_{2}/s=0.002$ for vertebrates and 0.003 for insects. Given the number of protein-coding genes we analyzed (21,208 for vertebrates and 15,283 for insects), we expect to find 52 ± 14 and 43 ± 13 genes with pCFMs in vertebrates and insects, respectively (95% confidence intervals are calculated using normal distribution approximation for binomial distribution with parameters *n* = 21,208, *P* = 0.002 and *n* = 15,283, *P* = 0.003). These values lie between the numbers of raw unfiltered indels (low-confidence data set, 468 genes in vertebrates and 156 in insects) and filtered indels (high-confidence data set, 5 and 1, respectively) that we obtain.

To model the evolution under the scenario of simultaneous occurrence of the two mutations (i.e., when the intermediate state is not observed), we additionally assume s=1, that is, that the intermediate state is lethal. In this case, we get ${Tm}_{1}m_{2}/s=1*10^{-5}$ for vertebrates and $4.5*10^{-6}$ for insects. This means we expect to find 1 ± 1 genes with pCFMs in vertebrates and 0 or 1 such gene in insects under this model.

The above models are simplistic, and make several assumptions that may not hold. In particular, we assume that the fixed pCFM confers the same fitness as the wild type sequence; if it is selectively inferior, the expected numbers will be lower, and if it confers a selective advantage, these numbers will be higher. Furthermore, we assume that the two mutations comprising the cPFM are independent of one another; with the double indel mutation happens at a higher rate than that expected on the basis of individual indel mutations, perhaps because of the variability in the rate of such mutations between genomic regions, the likelihood of pCFMs through both mechanisms will be increased, and the simultaneous fixation of both indels will be proportionally more likely.

Nevertheless, the above estimates suggest that the pCFMs in our data were most likely fixed via an intermediate uncompensated state.

## Conclusions

We describe 624 candidate pairs of compensatory frameshifting mutations in the past evolution of vertebrate and insect species, including six high-confidence cases that passed all our stringent filtering steps. We show that pCFMs tend to fix in poorly conserved segments in genes under relaxed negative selection, and the distribution of pCFMs along genes follows that of uncompensated indels. This suggests that pCFMs occur as a pair of distinct mutational events separated by a period of time, rather than a single mutational event of two simultaneous indels. This favors the model of origin of pCFMs under which a single deleterious frameshifting mutation segregates at a low frequency within a population until it is compensated by another indel mutation arising in the same haplotype. Overall, these results put forward pCFMs as a previously undescribed source of novelty in protein evolution.

## Materials and Methods

### Sequence Alignment

As a starting point, we used the 100-way vertebrate and 124-way insect (diptera) MULTIZ alignments obtained from the UCSC genome browser (Rosenbloom et al. 2015). These alignments comprised nonreference genomes being aligned to a reference (correspondingly, human or *Drosophila melanogaster*). This means that all the sites where the reference species has a deletion are absent, making these alignments unfit for our purposes. Therefore, we realigned the protein-coding sequences of genes from these species as follows. We extracted the exonic sequences from genomes using the annotation from the corresponding MULTIZ alignments, concatenated these exons into genes, and aligned the resulting genes using mafft v7 (Katoh et al. 2002). The following parameters were used both for vertebrate and for insects: –maxiterate 1,000 –globalpair –preservecase. To allow for detection of frameshifts, we performed a nucleotide-level, rather than a codon-level, alignment. In the insects data set, we removed the D_pseudoobscura_1 and A_gambiae_1 genomes as they were redundant (the alignment already contained the droPse3 and anoGam3 genomes from the same species). Because the gene annotation was based on the genome of the reference species, we excluded the overhanging 5′- and 3′-ends as well as long (>50 nucleotides) internal DNA segments in nonreference species that had no orthologous sequence in the reference. High-quality alignments were obtained for 21,208 out of 21,521 genes of vertebrates and 27,341 out of 30,482 genes in insects (insect genes comprised all NCBI RefSeq Gene CDS regions including nonprotein-coding ones; nonprotein-coding genes were excluded at subsequent filtering steps). The phylogenetic trees for vertebrates and insects obtained from whole-genome alignments, with branch lengths scaled in estimated numbers of nucleotide substitutions per site, were obtained from the UCSC genome browser.

### Inference of pCFMs

We designed an algorithm for detection of pCFMs using a multiple alignment and the corresponding phylogenetic tree.

In short, the algorithm uses a parsimony-based approach to detect insertions and deletions in the alignment. As the first step, it reconstructs past insertions and deletions that had occurred at specific phylogenetic branches, and are inherited by subsets of analyzed species. At the second step, to ensure usage of high-quality and high-confidence protein-coding sequences for pCFM inference, the nucleotide alignments for each gene were filtered on a sequence-by-sequence basis. We excluded the species that did not meet all of the following criteria: had the number of nucleotides divisible by 3; carried a start codon at the sequence start; carried a stop codon at the sequence end; carried no in-frame stop codons. At the third step, pCFMs are identified as pairs of indels of length not divisible by 3 in the remaining sequences; at this step, we also excluded sequences with more than two such indels, assuming that our reconstruction of pCFMs in such sequences would be less trustworthy. We performed quality filtering after indel classification, assuming that the added information from the presumably lower quality sequences allows for more robust classification of the evolutionary history of indels. For insect, data set alignments for which fewer than four sequences were left after filtering were not considered further; these included all nonprotein-coding genes.

For each detected pCFM, the algorithm outputs their type (insertions or deletions), positions, lengths, and the list of species they were detected in. A detailed description of the algorithm is provided in the supplementary text 1, Supplementary Material online (see also supplementary figs. 1 and 2, Supplementary Material online). The algorithm is implemented as a Python script, available at https://github.com/Captain-Blackstone/Compensatory-frameshifts (last accessed February 10, 2022).

### Postfiltering of pCFMs

With all the pCFMs detected, we then focused on the most reliable ones, narrowing down the considered sample as follows. First, although our algorithm detects pCFMs independently of whether they occurred in the same or in different branches of the phylogenetic tree, in this work, we only consider those that happened on the same branch. Second, we only considered pCFMs that comprised short indels, with combined lengths of no more than four nucleotides (i.e., two insertions of lengths 1 and 2; two deletions of lengths 1 and 2; an insertion and a deletion both of lengths 1; or an insertion and a deletion both of lengths 2). The reasoning behind this filter is that the probability of fixation of an indel rapidly declines with its length, whereas the probability of assembly artifacts may be expected to be less sensitive to indel length. Third, the remaining alignments were manually inspected, and only those with unambiguous pCFMs were retained. This resulted in 624 pCFMs, contained in 624 distinct genes.

To obtain the high-confidence data set, we focused on those cases where a pCFM was inherited by more than one species in our data set. This way, we could have excluded some bona fide species-specific pCFMs, but obtained additional support for the remaining ones, under the logic that coincident artifacts in different independently assembled genomes should be rare. We also excluded those alignments in which either the ancestral or the frameshifted sequence was not supported by running nBLAST (Altschul et al. 1990) against the genome of the corresponding species in the NCBI nr database.

This left us with ten pCFMs. For the vertebrate data set, we then sought additional support from other sequencing projects not included in the UCSC genome browser. For this, for each pCFM-carrying gene, we used ENSEMBL (Cunningham et al. 2019) to find orthologs in species that were more closely related to the pCFM-carrying species than any other species in our alignment. This rescued one additional pCFM for a total of 11 pairs.

To test the robustness of pCFM inference to alignment, we realigned each of these 11 pCFM-carrying genes using four other aligners: clustalw, t-coffee, muscle, and prank (all with default parameters). Only those pCFMs that were detected in each of the realignments were retained for the high-confidence data set, leaving us with six pCFMs.

### Statistical Approaches

For each gene, we used PAML version 4.8 (Yang 1997) to estimate the probabilities of nonsynonymous changes relative to synonymous ones (*ω* values) under the substitution model of Goldman and Yang (1994) described in Yang et al. (1998). We used the M0 model (model = 0 parameter) to estimate a single *ω* value for the entire tree, and a model with two *ω* parameters (model = 2 parameter) to fit distinct values of *ω* for the pre- and post-pCFM branches. The remaining parameters were the same in both models: runmode = 1, seqtype = 1, NSsites = 0. Each time we estimated *ω* for a gene with a pCFM, we excluded the region spanned by the pCFM from the alignment. This is because PAML treats gaps as “missing data” rather than as actual absence of the nucleotide. As a consequence, had these regions been retained, the codon boundaries in regions between frameshifting mutations would be wrong.

To ask whether the pCFM-carrying genes are characterized by increased or decreased *ω* values, we compiled a sample of non-pCFM-carrying genes as a control. The control sample was of the same size as the pCFM-carrying gene sample, with each pCFM-carrying gene having a non-pCFM-carrying gene pair. The pair was drawn randomly from non-pCFM-carrying genes, ensuring that the corresponding pCFM could have been detected by our algorithm if it were present in it. This was done by in silico insertion of a pCFM with the same parameters (lengths, types of indels, distance between them, species affected) in non-pCFM carrying gene with its further realignment.

For each amino acid site of the alignment, we calculated Shannon’s entropy as $H=-\sum_{i=1}^{n}P\left(x_{i}\right)ln\left(P\left(x_{i}\right)\right)$, where $P\left(x_{i}\right)$ is the proportion of *i*th amino acid in a given site.

For all conservation metrics (*ω*, phastCons score and Shannon’s entropy), we used as input only the subset of species that passed the filtering steps described in the “Inference of pCFMs” section, that is, those in which the considered gene started with a start codon, ended with a stop codon, had length divisible by 3, and had no internal stop codons. To test the robustness of conclusions to the choice of analyzed species, we also used two alternative approaches for picking species: 1) used all species in the alignment (“all” in supplementary table 4, Supplementary Material online); or 2) used a pair of species with reliable annotation: human and mouse for vertebrates, and *Drosophila melanogaster* and *Drosophila obscura* for insects; genes that did not pass all the quality filters in either of these two species were not analyzed (“two species” in supplementary table 4, Supplementary Material online).

To combine the signal across comparisons for individual genes, we used the Kolmogorov–Smirnov test with the null hypothesis of the uniform distribution of *P* values.

### Ancestral State Reconstructions for Nucleotide Sites

Ancestral states for individual nucleotide sites and phylogenetic positions of single-nucleotide substitutions were reconstructed independently of the inference of indels (see above) with the help of the maximum likelihood method implemented in MEGA6 (Tamura et al. 2013). For substitutions that had happened on the same phylogenetic branches as the pCFM, which one came first could not be established unambiguously. In these cases, we assumed that all substitutions occurred simultaneously; that both frameshifting indels in the pCFM occurred simultaneously; and considered two scenarios, assuming that the substitutions occurred either before or after the pCFM. For these branches, we identified the ancestral state as “*A*,” the intermediate state in the substitution-first scenario as “*A*_mut_,” the intermediate state in the frameshift-first scenario as “*A*_fr_,” and the derived state as “*E*.”

### Similarity Metrics

To study the effects of indels and single-nucleotide substitutions on the encoded amino acid sequence, we compared different states of these sequences using two similarity metrics. First, we measured the mean Miyata distance (Miyata et al. 1979) between the aligned amino acids in the pairwise alignment. This metric is also close to 0 for very similar sequences; for very distant sequences, it is close to 5.13, which is the maximum Miyata distance (namely, the distance between glycine and tryptophan). We considered gaps to have the maximum possible distance (5.13) to any amino acid. Second, we measured the difference between the mean hydropathy indexes of the two sequences (Kyte and Doolittle 1982). This difference is close to 0 for very similar sequences and close to 9 for very distant ones.

## Supplementary Material

Supplementary data are available at *Molecular Biology and Evolution* online.

## Supplementary Material

msac031_Supplementary_Data
